# Ammonia oxidation is not required for growth of Group 1.1c soil Thaumarchaeota

**DOI:** 10.1093/femsec/fiv001

**Published:** 2015-01-14

**Authors:** Eva B. Weber, Laura E. Lehtovirta-Morley, James I. Prosser, Cécile Gubry-Rangin

**Affiliations:** Institute of Biological and Environmental Sciences, Cruickshank Building, St Machar Drive, University of Aberdeen, Aberdeen, AB24 3UU, UK

**Keywords:** acidic forest, Thaumarchaeota, soil archaea, temperature, organic carbon, organic nitrogen

## Abstract

Thaumarchaeota are among the most abundant organisms on Earth and are ubiquitous. Within this phylum, all cultivated representatives of Group 1.1a and Group 1.1b Thaumarchaeota are ammonia oxidizers, and play a key role in the nitrogen cycle. While Group 1.1c is phylogenetically closely related to the ammonia-oxidizing Thaumarchaeota and is abundant in acidic forest soils, nothing is known about its physiology or ecosystem function. The goal of this study was to perform *in situ* physiological characterization of Group 1.1c Thaumarchaeota by determining conditions that favour their growth in soil. Several acidic grassland, birch and pine tree forest soils were sampled and those with the highest Group 1.1c 16S rRNA gene abundance were incubated in microcosms to determine optimal growth temperature, ammonia oxidation and growth on several organic compounds. Growth of Group 1.1c Thaumarchaeota, assessed by qPCR of Group 1.1c 16S rRNA genes, occurred in soil, optimally at 30°C, but was not associated with ammonia oxidation and the functional gene *amoA* could not be detected. Growth was also stimulated by addition of organic nitrogen compounds (glutamate and casamino acids) but not when supplemented with organic carbon alone. This is the first evidence for non-ammonia oxidation associated growth of Thaumarchaeota in soil.

## Abstract (precis)

Uncultivated soil Group 1.1c Thaumarchaeota are abundant, but have no known function. We report their growth without ammonia oxidation, unlike thaumarchaeal relatives, and stimulation by organic C.

## Graphical abstract

**Figure:**
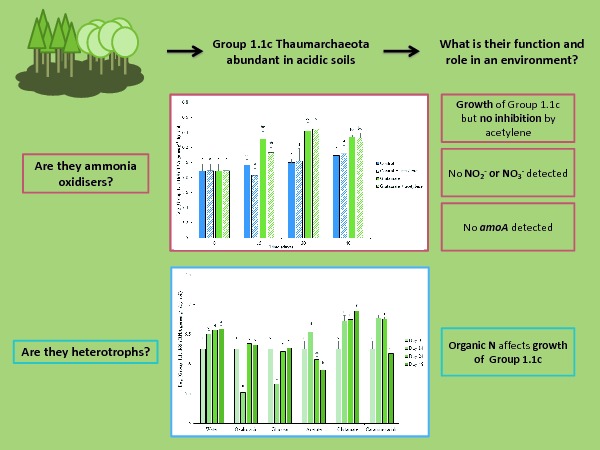
Uncultivated soil Group 1.1c Thaumarchaeota are abundant, but have no known function. We report their growth without ammonia oxidation, unlike thaumarchaeal relatives, and stimulation by organic C.

## INTRODUCTION

Traditionally, archaea were considered to be extremophiles, inhabiting ecosystems with very low pH, low oxygen concentration, high temperature or high salt concentration. The advent of molecular techniques led to two ground-breaking studies in which 16S rRNA sequences representative of a major archaeal lineage, the Crenarchaeota, were detected in cold seawater sediments (DeLong 1992; Fuhrman, McCallum and Davis 1992). This novel non-extremophilic group was only distantly related to cultivated crenarchaeal extremophiles and was named Group 1 Crenarchaeota. Subsequent studies confirmed the abundance and ubiquity of non-thermophilic Group 1 Crenarchaeota in other non-extreme environments, including soil (Ochsenreiter *et al.*, 2003). More recent detailed analysis of archaeal genomes placed Group 1 Crenarchaeota in a newly defined phylum, the Thaumarchaeota (Brochier-Armanet *et al.*, 2008). The Thaumarchaeota consist of several clusters, for which delineation is based on the 16S rRNA gene only (e.g. pSL12, ALOHA and Group 1.1c), while classification of Groups 1.1a, 1.1b, SAGMGC-1 and HWCGIII is based on several genes (16S rRNA and *amoA* genes) (Pester, Schleper and Wagner 2011).

Within the Thaumarchaeota, Groups 1.1a and 1.1b have been studied most intensively and are common in marine and soil environments, respectively (Leininger *et al.*, 2006; Auguet, Barberan and Casamayor 2010). Currently, there are 25 cultivated members of the Thaumarchaeota, all of which can oxidize ammonia to nitrite, and 12 representative genomes, all of which possess homologues of genes encoding the key functional enzyme ammonia monooxygenase (see Lehtovirta-Morley *et al.*, 2014 and references therein). Ammonia oxidation is the first and rate-limiting step of nitrification and is performed by ammonia-oxidizing bacteria (AOB) and archaea (AOA). Although both AOB and AOA are important for nitrification in terrestrial ecosystems, AOA appear to contribute more to nitrification in acidic soil environments (Nicol *et al.*, 2008; Gubry-Rangin, Nicol and Prosser 2010; Stopnišek *et al.*, 2010).

Thaumarchaeal Group 1.1c sequences were first reported in Finnish boreal soil (Jurgens, Lindström and Saano 1997) and phylogenetic analysis of 16S rRNA genes showed their close relation to Groups 1.1a and 1.1b, despite their higher GC content than other Group 1 Thaumarchaeota (Delong 1998; Nicol *et al.*, 2007). Subsequent studies indicated that Group 1.1c 16S rRNA sequences are abundant in acidic forest soils (Jurgens, Lindström and Saano 1997; Bomberg and Timonen 2009), representing up to 29% of all 16S rRNA genes sequences in one sample core from a mixed deciduous forest (Kemnitz, Kolb and Conrad 2007). Group 1.1c represented 29% of total thaumarchaeal clones in an acidic forest peat soil (Stopnišek *et al.*, 2010) and 34% of all archaeal sequences in a tropical savannah soil (Catão *et al.*, 2013). Furthermore, these organisms accounted for up to 1.8% of total Thaumarchaeota in an acidic agricultural soil (pH range 4.5–6.0) (Lehtovirta, Prosser and Nicol 2009). Group 1.1c Thaumarchaeota have also been detected in soils from high altitudes, such as montane acidic forest soil (Oline, Schmidt and Grant 2006), mature soils in glacier foreland (Nicol *et al.*, 2006), high mountain lakes (Auguet and Casamayor 2013), hot springs (Bohorquez *et al.*, 2012) and tropical forest soils (Tripathi *et al.*, 2013). Finally, thaumarchaeal Group 1.1c populations were detected in the rhizosphere of pine trees colonized by ectomycorrhizal fungi in boreal forest soils (Bomberg and Timonen 2009; Bomberg *et al.*, 2011).

The predominance of Group 1.1c Thaumarchaeota in soil and other environments suggests an important ecosystem function, with suggested involvement in metabolism of C1 compounds (Kemnitz, Kolb and Conrad 2007; Bomberg, Montonen and Timonen 2010). Assessment of the ecosystem function of Group 1.1c Thaumarchaeota is, however, severely limited by difficulty in obtaining enrichments or pure cultures of representatives of this group for physiological characterization. The aim of this study was to gain information on the *in situ* physiology of Group 1.1c Thaumarchaeota in soil by assessing their growth under a range of conditions in controlled soil microcosms and, in particular, to assess whether they, like other well-characterized Thaumarchaeota, are involved in ammonia oxidation.

## MATERIALS AND METHODS

### Primer design and PCR conditions

Primers specifically targeting Group 1.1c Thaumarchaeota and excluding Groups pSL12, ALOHA, 1.1a, SAGMGC-1 and 1.1b were designed for use in PCR and quantitative PCR (qPCR). Primers were designed by acquiring representative 16S rRNA gene sequences of all major thaumarchaeal groups in the GenBank database derived from clone libraries generated from soil DNA in previous studies (Table S1, Supporting Information). Sequences were aligned using ClustalW (Thompson, Higgins and Gibson 1994), implemented in BioEdit software (Hall 1999). Several primer sets were designed using Geneious software (Biomatters Limited, New Zealand) and further tested by alignment against thaumarchaeal sequences to confirm their specificity for the Group 1.1c. The specificity of primers was verified by PCR amplification of cultures from 1.1a, 1.1b, SAGMGC-1 groups and a Group 1.1c clone obtained from the study of Lehtovirta, Prosser and Nicol (2009). The primer set with the highest specificity for Group 1.1c Thaumarchaeota was 318F (5^′^-CCTTGAGAGAGGKGGCCCGG-3^′^) and 385R (Lehtovirta, Prosser and Nicol 2009), resulting in an amplicon of 109 bp. PCR amplification using this primer set was performed in a 50-μL reaction using 1 μL of undiluted DNA, 1xPCR buffer, 1.5 mM MgCl_2_, 200 nM each primer, 200 nM bovine serum albumin, 1 mM dNTPs and 1 U Bio*Taq* polymerase (Bioline, London, UK). Cycling conditions were 1 cycle of 95°C for 5 min, 35 cycles of 94°C for 30 s, 55°C for 30 s, 72°C for 45 s and 1 cycle of 72°C for 10 min.

### Soil sampling and nucleic acid extraction

Ten acidic soil-sampling sites were selected within the Scottish Highlands with a range of vegetation (pine, birch or grassland), using the National Soil Inventory of Scotland (NSIS) (Table 1). Five soil samples were collected in May (average seasonal temperature in Scotland = 10°C) and an additional soil sample of Lower Dell soil was taken in September 2014 (average seasonal temperature in Scotland = 13°C). Soil was sampled randomly at each site from the upper 30 cm of the soil profile using a corer. Samples from the same site were later pooled and sieved through a 3.5-mm metal sieve. DNA was extracted and gene abundance were determined (see below) immediately after return to the laboratory (less than 24 h), and soil for use in microcosm experiments was stored at 4°C until further use. Moisture content was determined after drying the soil sample for 24 h at 105°C and soil pH was measured in water (1:5 w/v) after 30 min of shaking followed by 10 min of resting at room temperature. Ammonium concentration and combined nitrite plus nitrate concentration were determined using colorimetric flow injection analysis (FIA star 5000 Analyzer, Foss Tecator) after extraction in 1M KCl (1:2, w/v). Nucleic acids were extracted from 0.5 g of soil as previously described (Griffiths *et al.*, 2000). The presence of AOA and AOB was assessed by PCR amplification of respective *amoA* genes from extracted nucleic acids using primer sets 23F, 616R (Tourna *et al.*, 2008) and 1F, 2R (Rotthauwe, Witzel and Liesack 1997) under conditions previously described by Nicol *et al.* (2008).

**Table 1.** Location and characteristics of soil sampling sites. ND—not determined.

Sampling site	Grid reference (easting, northing)	Vegetation	Moisture (%)	pH	Group 1.1c detected (Group 1.1c gene abundance g^−1^ dry soil)	AOA detected	AOB detected
Wester Manbeen	320 000, 860 000	Birch forest	12.4	6.14	2.1 × 10^5^	Yes	Yes
Dochfour	259 935, 839 930	Birch forest	9.0	4.65	ND	Yes	No
Rathens	400 029, 859 999	Grassland	27.9	5.65	2.4 × 10^5^	Yes	Yes
Windy Hills	380 001, 839 931	Pine forest	40.4	3.92	6.6 × 10^5^	Yes	No
Drumguish	280 029, 800 014	Pine forest	39.0	3.70	8.8 × 10^5^	No	No
Culbin	298 797, 861 766	Pine forest	11.1	5.86	ND	No	No
Fersit	239 975, 780 003	Pine forest	33.4	4.20	2.5 × 10^6^	No	No
Lower Dell	299 995, 820 004	Pine forest	25.0	3.80	3.6 × 10^6^	No	No
Kintessack	300 017, 860 012	Pine forest	24.5	4.10	ND	Yes	No
Edradour	299 998, 760 000	Pine forest	29.6	4.60	ND	Yes	No

### Microcosm incubations

Separate sets of microcosms were constructed containing each of the soils in which Group 1.1c Thaumarchaeota could be detected using the above PCR assay (Table 1). Microcosms consisted of 10 g of soil in 125-ml serum bottles in triplicate for each treatment. Bottles were closed with a rubber cap and were aerated every 3–4 days to ensure aerobic conditions during incubation at three temperatures (10, 20 and 30°C) for 30 days, to provide information on optimal growth temperature.

To determine the potential for autotrophic nitrification, a second experiment was performed, using only Lower Dell soil, based on the results of the first experiment. Microcosms were amended with 20 mM glutamate (final concentration of 200 μg N g^−1^ dry soil), to provide a source of organic nitrogen that would generate inorganic ammonium through mineralization, and also provide organic carbon. Acetylene (0.01%, v/v), an inhibitor of both archaeal and bacterial ammonia oxidizers (Offre, Prosser and Nicol 2009; Gubry-Rangin, Nicol and Prosser 2010), was added to one set of microcosms to observe its effect on Group 1.1c growth. Two sets of control (water-amended) microcosms were also used, either with or without acetylene. Microcosms were incubated for 40 days at 30°C.

In a third experiment, the effect of additional organic sources on Group 1.1c Thaumarchaeota was studied by amendment of microcosms with 1 ml of casamino acids (1 g l^−1^) or of 0.1 M glutamate, acetate, glucose or oxalic acid solution, and incubation for 48 days at 30°C to test the effect of organic compounds with or without nitrogen.

For all three experiments, net nitrogen mineralization and ammonia oxidation were determined by measuring temporal changes in ammonia and nitrite plus nitrate concentrations and pH during incubation.

### Estimation of gene abundance

The abundance of Group 1.1c Thaumarchaeota was estimated by qPCR of 16S rRNA genes using primers 318F and 385R. Standards for qPCR were prepared using a Group 1.1c thaumarchaeal clone (Lehtovirta, Prosser and Nicol 2009) in a series of dilutions with abundance 10^8^ to 10^1^ per reaction. The assay was performed in a 20-μl reaction containing 2 μl of extracted DNA (diluted to 2 ng μl^−1^), 400 nM of each primer, 1% (v/v) formamide and 10.5 μl QuantiTect SYBR Green PCR Master Mix (Qiagen, Crawley, UK). Cycling conditions were 1 cycle of 95°C for 15 min, 40 cycles of 94°C for 10 s and 60°C for 30 s, followed by measurement of fluorescence at 72°C for 8 s. Finally, a melting curve measurement was performed at a 0.2°C interval for 20 min, from 60°C to 95°C and each amplified product was run on a 1% agarose gel. All assays had 90–95% efficiency and an R^2^-value of 0.998–1.

The abundance of all Thaumarchaeota was estimated using primers 771F and 957R (Ochsenreiter *et al.*, 2003). Standards for qPCR were prepared as described above and the assay was performed in a 20-μl reaction containing 2 μl of extracted DNA (diluted to 2 ng μl^−1^), 400 nM of each primer and 10.5 μl QuantiTect SYBR Green PCR Master Mix, with the same cycling conditions as described above.

### Statistical analysis

To determine the effect of different treatments and time on Group 1.1c 16S rRNA gene abundance and ammonium concentration, means from triplicate microcosms were compared using two-way ANOVA in SigmaPlot (Systat Software, Inc., UK) and the Fisher's least significant difference *post-hoc* test was performed to compare means of each condition. Data were log-transformed to ensure that normal distribution and homogeneity of variance and significance was assessed at *P* < 0.05.

## RESULTS

### Abundance and growth of Group 1.1c Thaumarchaeota

**Figure 1.**
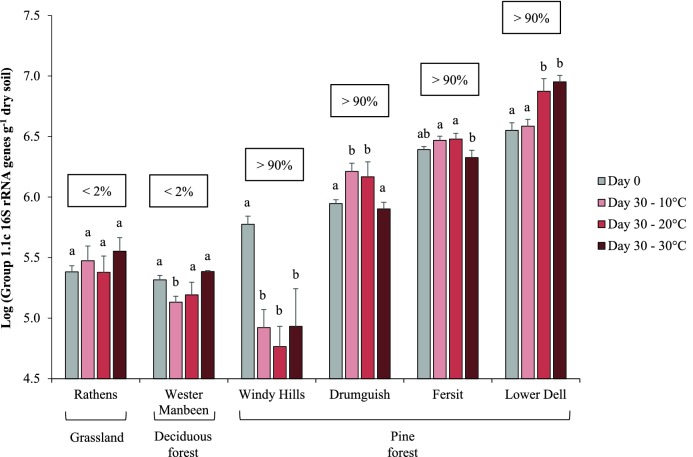
Abundance of Group 1.1c 16S rRNA genes in six soil microcosms incubated at three temperatures for 30 days. Data are presented as mean and standard error of triplicate microcosms for each treatment. Different letters above the bars represent significant difference in abundance (*P* < 0.05) based on a two-way ANOVA. Numbers within the squares represent the proportion of Group 1.1c Thaumarchaeota relative to total thaumarchaeal abundance (based on respective 16S rRNA gene abundances).

Group 1.1c Thaumarchaeota were detected in six out of ten soils sampled using a qPCR assay targeting Group 1.1c 16S rRNA with a detection limit of 10^4^ genes g^−1^ dry soil (Table 1). In these soils, Group 1.1c Thaumarchaeota represented approximately 2% of the thaumarchaeal community in grassland and birch forest soils and 90% in pine tree forest soils. Microcosms were constructed containing these to investigate the growth of Group 1.1c Thaumarchaeota (estimated by an increase in Group 1.1c 16S rRNA gene abundance by qPCR). Growth of Group 1.1c Thaumarchaeota was not detected in soils with low abundance (<10^4^ genes g^−1^ dry soil) (Wester Manbeen, Rathens, Windy Hills) and, surprisingly, also in one soil with a relatively high initial abundance (Fersit) (>10^6^ genes g^−1^ dry soil) after incubation for 30 days at any of the three temperatures used (Fig. 1). Gene abundance increased significantly in the two remaining soils, Drumguish and Lower Dell, both of which are pine tree forest soils with high Group 1.1c initial abundance (>10^6^ genes g^−1^ dry soil) (Fig. 1). Detection of growth was temperature-dependent and occurred in Drumguish soil at 10 and 20°C (1.88- and 1.79-fold increases, respectively, after incubation for 30 days) and at 20 and 30°C in Lower Dell soil (2.17- and 2.50-fold increases, respectively). In these two soils, where the abundance of Group 1.1c 16S rRNA genes was greatest, neither AOA nor AOB *amoA* genes could be detected using PCR methods with a detection limit of 10^2^ copies reaction^−1^. We believe that the inability to detect Group 1.1c genes in these two soils is not due to methodological limitations, as AOA and AOB were detected in six and two soils, respectively (Table 1). Because Group 1.1c thaumarchaeal abundance and growth were greatest in Lower Dell soil incubated at 30°C, this soil and temperature were selected for subsequent microcosm studies.

### Assessing potential autotrophic ammonia oxidation by Group 1.1c Thaumarchaeota

To determine whether growth of Group 1.1c Thaumarchaeota is associated with autotrophic ammonia oxidation, microcosms containing Lower Dell soil were incubated after amendment with glutamate in the presence and absence of acetylene. Ammonium increased significantly in control microcosms, indicating mineralization of existing organic nitrogen, producing 90 μg NH_4_^+^-N g^−1^ dry soil during incubation for 40 days (Fig. S1, Supporting Information). Ammonium concentration was significantly greater in glutamate-amended microcosms, reaching 237 μg NH_4_^+^-N g^−1^ dry soil (non-acetylene treated) after incubation for 15 days, indicating mineralization of an important proportion of the added glutamate (Fig. S1, Supporting Information). Acetylene did not significantly affect ammonium concentration in either control or glutamate-amended soils, indicating no significant ammonium loss through nitrification. In addition, nitrite plus nitrate concentration remained below the limit of detection (3 μg (NO_2_^−^ + NO_3_^−^)-N g^−1^ dry soil) in all samples, again providing no evidence for nitrification under these conditions.

**Figure 2.**
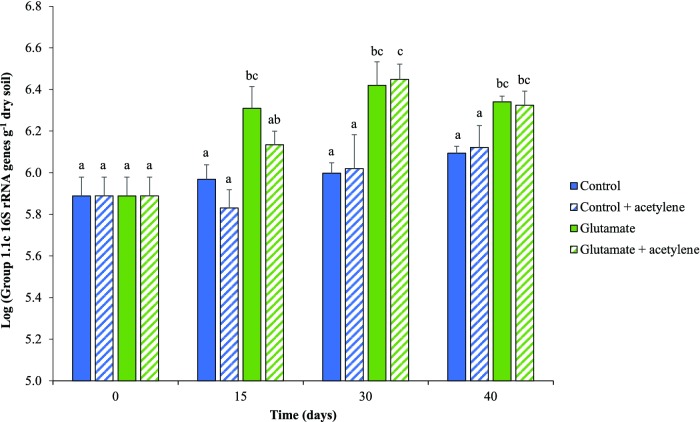
Temporal changes in abundance of Group 1.1c 16S rRNA genes in microcosms amended with water or glutamate and/or acetylene and incubated at 30°C for 40 days. Data are presented as mean and standard error of triplicate microcosms for each treatment. Different letters above the bars represent significant differences in abundance (*P* < 0.05), based on a two-way ANOVA.

Growth of Group 1.1c Thaumarchaeota was not significant in control microcosms (Fig. 2), with gene abundances lower than observed previously (Fig. 1), presumably due to storage of soil at 4°C for 2 months. However, Group 1.1c Thaumarchaeota did grow in glutamate-amended microcosms during the first 15 days of incubation, with no subsequent significant increase in Group 1.1c 16S rRNA gene abundance; this indicates relatively rapid growth of Group 1.1c Thaumarchaeota in soil (Fig. 2). Again, acetylene did not significantly affect growth in control microcosms (Fig. 2) and neither AOA nor AOB *amoA* genes could be detected in any microcosm sample.

### Effect of different organic carbon sources on Group 1.1c thaumarchaeal growth

To determine whether carbon sources other than glutamate could increase the abundance of Group 1.1c Thaumarchaeota, microcosms were amended with each of five organic compounds. Again there was no evidence of nitrification, and nitrite plus nitrate concentration was below the level of detection in all microcosms.

Ammonium production through mineralization increased in all microcosms during the first 15 days of incubation, except those amended with glucose. Glucose presumably stimulated assimilation, in comparison to control microcosms, and ammonium concentration only reached 20 μg NH_4_^+^-N g^−1^ dry soil after incubation for 15 days (compared to 69 μg NH_4_^+^-N g^−1^ dry soil in control microcosms) and decreased after incubation for 24 days (Fig. S2, Supporting Information). Ammonium production was greatest following mineralization of casamino acids (1.7 mg NH_4_^+^-N g^−1^ dry soil). As observed previously with glutamate, mineralization of all organic compounds (except glucose) occurred within the first 15 days of incubation, after which ammonium concentration did not change significantly (Fig. S2, Supporting Information).

**Figure 3.**
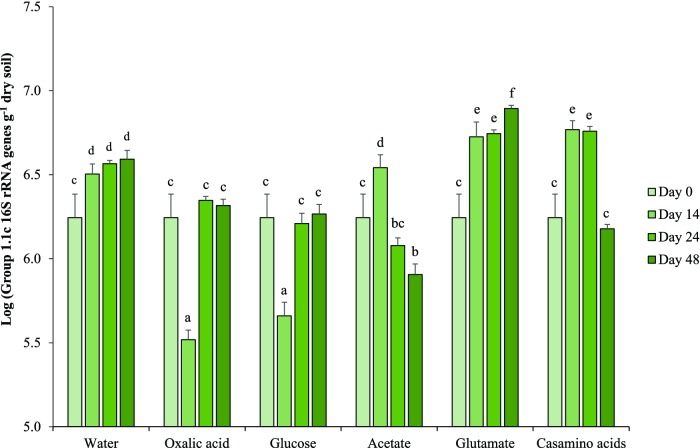
Temporal changes in abundance of Group 1.1c 16S rRNA gene in soil microcosms supplemented with different organic sources and incubated for 48 days. Data are presented as mean and standard error of triplicate microcosms for each treatment. Different letters above the bars represent significant differences in abundance (*P* < 0.05), based on a two-way ANOVA.

Amendment of soil with different carbon sources had varying effects on Group 1.1c thaumarchaeal growth (Fig. 3). Growth again occurred in control microcosms and was stimulated in glutamate-amended microcosms, with a 4-fold increase in Group 1.1c 16S rRNA gene abundance after incubation for 48 days. Casamino acids and acetate led to 3- and 2-fold increases in gene abundance during the first 14 days of incubation, respectively, but abundance subsequently decreased to or below initial abundance, respectively. Amendment with glucose or oxalic acid induced a significant decrease in gene abundance during the first 14 days of incubation, which was followed by a significant increase and restoration of initial abundance. Again, AOA and AOB *amoA* genes were below the detection level in all microcosm samples. For all three experiments, no pH changes were observed over the course of incubation.

## DISCUSSION

Group 1.1c Thaumarchaeota were detected in several Scottish soil ecosystems and Group 1.1c thaumarchaeal 16S rRNA gene relative abundance ranged from 2% of total thaumarchaeal abundance, in grassland and birch forest soils, to 90% of the thaumarchaeal community in pine forest soils. Tripathi *et al.*, (2013) also reported greater abundance of Group 1.1c in forest soils than in non-forest soils, while, in contrast, Yarwood, Bottomley and Myrold (2010) observed similar Group 1.1c abundance in deciduous and pine forest soils (Yarwood, Bottomley and Myrold 2010). Group 1.1c thaumarchaeal sequences have also been detected previously in mildly acidic agricultural soil (pH 6) (<5 × 10^4^ genes g^−1^ dry soil) (Lehtovirta, Prosser and Nicol 2009), but at a much lower abundance than observed here in a grassland soil of the same pH (4 × 10^5^ genes g^−1^ dry soil). These comparisons must, however, be interpreted with caution due to the use of different primers and assay conditions, which may introduce biases. Nevertheless, the high abundance of Group 1.1c Thaumarchaeota detected in acidic pine tree soils suggests their preference for and potential adaptation to this environment and is consistent with previous studies (e.g. Bomberg and Timonen 2009).

A major aim of this study was identification and design of experimental conditions for growth of Group 1.1c Thaumarchaeota, by varying temperature and organic carbon amendments, and led to the first demonstration of growth, to our knowledge, of Group 1.1c Thaumarchaeota in soil microcosms. Incubation temperatures followed those used by Tourna *et al.*, (2008), who reported optimal growth of ammonia-oxidizing Thaumarchaeota at 30°C in microcosms containing a pH 7, agricultural soil. Optimal temperatures for growth of Group 1.1c Thaumarchaeota varied with the different soils investigated, with no obvious relation to soil type.

This study also investigates whether Group 1.1c oxidize ammonia as all cultivated Thaumarchaeota can grow autotrophically, gaining energy from the oxidation of ammonia to nitrite. There was no evidence of ammonia oxidation during growth of Group 1.1c Thaumarchaeota in soil microcosms. In addition to inorganic ammonia present in soils, ammonia was produced through mineralization in microcosms amended with glutamate, which was previously shown to be a preferential source of ammonia for AOA in restored peatland and agricultural soils (Levičnik-Höfferle *et al.*, 2012; Chan, McCormick and Ma 2013). Ammonium increased significantly through mineralization of natural or added organic nitrogen, but there was no evidence of its oxidation to nitrite or nitrate. In addition, there was no evidence of a decrease in ammonia concentration and the rate of ammonium accumulation was unchanged in microcosms amended with acetylene, an inhibitor of bacterial and archaeal ammonia oxidation (Offre, Prosser and Nicol 2009). Finally, neither archaeal nor bacterial *amoA* genes were detected in any microcosm using primers that have previously been successful in targeting *amoA* genes in cultivated AOA or in environmental DNA. The possibility of novel *amoA* genes in Group 1.1c Thaumarchaeota that would not be detected by these primers cannot be ruled out, but there was no evidence that growth of Group 1.1c Thaumarchaeota in any of the soils studied was associated with ammonia oxidation.

Stimulation of Group 1.1c thaumarchaeal growth following amendment of microcosms with organic compounds suggests heterotrophic growth, but those used in this study are readily available and utilizable by many members of the indigenous microbial community within a short period after addition. Significant growth of Group 1.1c Thaumarchaeota by direct metabolism of these compounds is therefore unlikely to have led to all of the observed growth, as abundance continued to increase for several weeks. Growth is more likely to have proceeded through indirect utilization of associated by-products and/or stimulation of other members of the community. Nevertheless, stimulation was greatest following amendment with compounds that provide a source of organic nitrogen (glutamate and casamino acids) and was not observed at all after addition of glucose. Stimulation of thaumarchaeal ammonia oxidizers by casamino acids has been demonstrated in a pure culture of *Nitrosotalea devanaterra* (Lehtovirta-Morley *et al.*, 2014) and by glutamate in soil (Levičnik-Höfferle *et al.*, 2012), and has been attributed solely to provision of ammonia by mineralization (Stopnišek *et al.*, 2010). Stimulation of Group 1.1c Thaumarchaeota by organic nitrogen suggests the potential for other metabolic processes, but requires physiological analysis of pure cultures. When amending microcosms with organic carbon alone, Group 1.1c growth seemed to be suppressed, presumably by other microbial communities in soil. In acetate-amended microcosms, an increase in Group 1.1c gene abundance was observed in the first 14 days. Acetate is converted to acetic acid below pH 5.5 and since observed growth is not significantly higher than in control microcosms, it might indicate adaptation or tolerance of Group 1.1c to high concentrations of acetic acid. Any adaptation was temporary, as Group 1.1c gene abundance decreased after 24 days of incubation. Mixotrophic growth of cultivated AOA has been observed (Tourna *et al.*, 2008; Lehtovirta-Morley *et al.*, 2014), there is evidence for assimilation of amino acids by marine ammonia-oxidizing Thaumarchaeota (Ouverney and Fuhrman 2000; Ingalls *et al.*, 2006) and ammonia oxidation could not be detected in a wastewater treatment system dominated by Group 1.1b archaea (Mußmann *et al.*, 2011). Thaumarchaeal growth has, however, only previously been observed in association with ammonia oxidation or *amoA* genes. We cannot rule out the possibility of undiscovered *amoA* genes that are undetectable using current primers, or specific conditions required for ammonia oxidation, but using methods and conditions routinely used for detection of ammonia oxidation and *amoA* genes, Group 1.1c Thaumarchaeota grew with no evidence of ammonia oxidation and suggests a different ecosystem function for this sister group of ammonia-oxidizing Thaumarchaeota.

In conclusion, this study demonstrates widespread distribution and high abundance of Group 1.1c Thaumarchaeota in several soils and provides a range of conditions under which growth of these organisms is favoured. Their growth in soil was stimulated by organic nitrogen but there was no evidence for associated ammonia oxidation, providing strong evidence for heterotrophic growth of Group 1.1c Thaumarchaeota in soil. Growth was stimulated by the addition of organic compounds containing nitrogen, but not by organic carbon alone, but it is not possible to determine whether stimulation was due to direct effects of added compounds, or secondary effects on the indigenous microbial community. This study therefore provides the first evidence of heterotrophic, non-ammonia-oxidation-associated growth of Thaumarchaeota in soil and provides the basis for isolation of representatives of this group in laboratory culture for more detailed physiological analysis.

## SUPPLEMENTARY DATA

Supplementary data is available at FEMSEC online.

Supplementary data is available at FEMSEC online
