# Supplementary material for: Ammonia oxidation is not required for growth of Group 1.1c soil Thaumarchaeota
Source: FEMS Microbiol Ecol. 2015 Jan 14;91(3):fiv001. doi: 10.1093/femsec/fiv001 (PMC4399444; doi:10.1093/femsec/fiv001)
Supplement: Supplementary data is available at FEMSEC online [file femsec_fiv001_index.html]

SUPPLEMENTARY DATA | FEMS Microbiology Ecology

## SUPPLEMENTARY DATA

**Files in this Data Supplement:**

- SUPPLEMENTARY DATA
- SUPPLEMENTARY DATA
