# Supplementary material for: Ammonia oxidation is not required for growth of Group 1.1c soil Thaumarchaeota
Source: FEMS Microbiol Ecol. 2015 Jan 14;91(3):fiv001. doi: 10.1093/femsec/fiv001 (PMC4399444; doi:10.1093/femsec/fiv001)
Supplement: Supplementary data is available at FEMSEC online [file FigureS1.pdf]

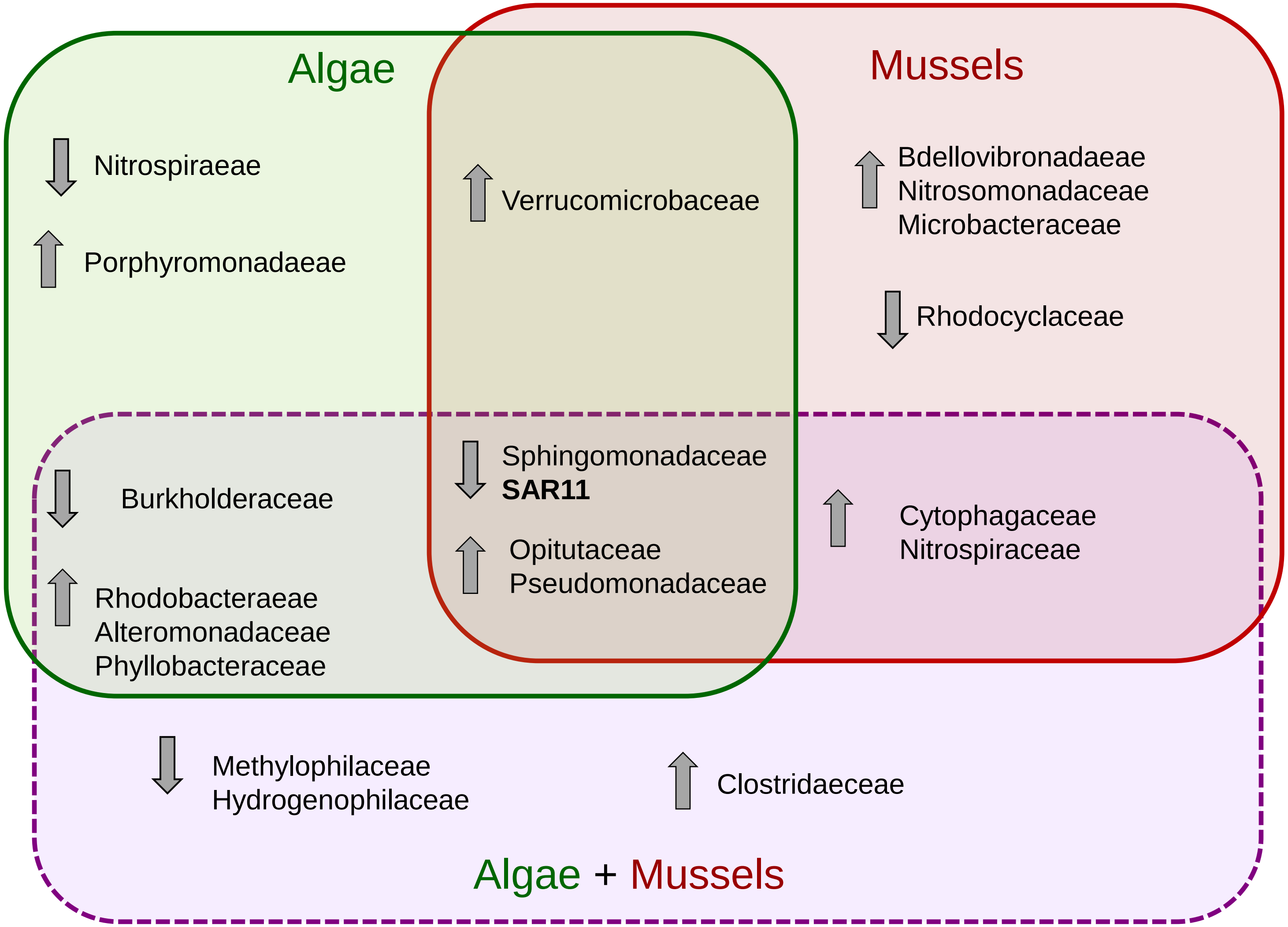

Supplementary Figure S1. Changes in bacterial families in day 21 microcosm libraries relative to day 21 control microcosm libraries, with common and distinct changes for the three addition treatments indicated. Families which changed significantly (DESeq analysis, $P < 0.05$) are grouped as increasing or decreasing relative to control and genera in bold appeared or disappeared relative to controls.
