# Supplementary material for: Ammonia oxidation is not required for growth of Group 1.1c soil Thaumarchaeota
Source: FEMS Microbiol Ecol. 2015 Jan 14;91(3):fiv001. doi: 10.1093/femsec/fiv001 (PMC4399444; doi:10.1093/femsec/fiv001)
Supplement: Supplementary data is available at FEMSEC online [file TabelS1_2.pdf]

Table S1. List of all genera identified in microcosm libraries with abundance >0.05% of sequences identified to family, with mean sequence abundance over the triplicate libraries (standard deviation) for each treatment. IS – incertae sedis.
Total abundance is % of sequences identified to family.

				Control		Algae		Mussels		Algae+Mussels		Control		Algae		Mussels		Algae+Mussels			
**Family**	**Genus**	**Day 0**		**Day 7**		**Day 7**		**Day 7**		**Day 7**		**Day 21**		**Day 21**		**Day 21**		**Day 21**			% total
		Mean	SD	Mean	SD	Mean	SD	Mean	SD	Mean	SD	Mean	SD	Mean	SD	Mean	SD	Mean	SD	**Totals**	abundance
Acidobacteria_Gp4	Gp4	974	122	479	261	386	95	495	417	806	273	741	320	712	273	746	398	841	298	8635	22.80
Burkholderiales_IS	Methylibium	470	71	451	266	461	134	394	178	392	152	387	160	314	105	320	156	270	77	4758	12.56
Nitrospiraceae	Nitrospira	171	43	119	56	99	29	139	80	195	79	213	91	163	68	287	148	312	109	2404	6.35
Chitinophagaceae	Terrimonas	223	29	162	89	169	54	154	72	139	45	104	39	117	53	124	50	124	49	1797	4.74
Acidobacteria_Gp6	Gp6	146	20	100	54	113	44	109	49	137	59	134	50	127	61	128	47	124	58	1560	4.12
Burkholderiales_IS	Ideonella	96	15	109	66	123	51	114	50	90	32	68	25	71	34	69	28	56	21	1118	2.95
Chitinophagaceae	Ferruginibacter	117	8	99	62	110	37	86	36	83	32	60	21	85	46	66	19	68	36	1073	2.83
Gemmatimonadaceae	Gemmatimonas	46	13	66	36	65	27	73	34	51	13	52	27	57	23	40	17	45	21	707	1.87
Comamonadaceae	Curvibacter	52	15	73	46	85	31	66	26	39	13	36	20	61	28	38	10	37	18	695	1.83
Opitutaceae	Opitutus	21	5	36	21	47	19	55	26	39	18	34	17	62	30	53	19	79	43	625	1.65
Flavobacteriaceae	Flavobacterium	74	12	30	13	56	34	50	19	54	32	24	8	42	30	24	7	41	42	591	1.56
Comamonadaceae	Hydrogenophaga	37	5	51	33	74	42	62	14	40	20	37	24	43	16	31	12	25	10	576	1.52
Spartobacteria	Spartobacteria_gen_IS	35	22	69	103	53	45	22	16	25	16	13	2	21	29	29	23	22	17	561	1.48
Rhodocyclaceae	Dechloromonas	29	9	34	21	74	75	28	31	32	5	19	11	43	65	23	28	19	9	557	1.47
Hydrogenophilaceae	Thiobacillus	46	25	44	24	28	3	37	25	35	11	36	17	33	20	35	17	25	9	470	1.24
Comamonadaceae	Rhodoferax	29	3	26	22	89	93	41	34	28	10	17	8	16	7	10	3	13	8	457	1.21
Acidobacteria_Gp17	Gp17	48	9	21	9	25	12	25	11	32	14	32	13	40	20	30	8	37	20	405	1.07
Sphingomonadaceae	Novosphingobium	39	14	30	11	22	7	16	13	24	8	57	37	34	6	18	9	23	9	377	1.00
Acidobacteria_Gp22	Gp22	36	10	34	17	30	11	29	23	30	10	21	7	21	10	29	13	21	6	358	0.95
Sinobacteraceae	Steroidobacter	34	19	30	11	27	11	25	14	32	23	21	5	28	19	20	3	17	10	348	0.92
Cyanobacteria_Family II	GpIIa	2	3	12	7	7	1	14	21	6	10	45	34	58	35	34	27	9	12	337	0.89
Sphingomonadaceae	Sphingobium	3	3	20	17	6	8	16	9	9	5	71	62	29	25	13	9	14	4	321	0.85
Sphingomonadaceae	Sphingopyxis	31	11	23	9	17	6	19	8	18	8	17	8	22	12	21	8	28	13	279	0.74
Hyphomicrobiaceae	Hyphomicrobium	35	8	13	6	13	6	13	5	20	10	26	13	29	13	21	6	21	10	268	0.71
Rhodobacteraceae	Rhodobacter	22	8	11	2	12	7	13	5	12	5	15	9	37	36	21	11	24	11	261	0.69
Saprospiraceae	Haliscomenobacter	16	5	26	16	24	6	20	10	19	7	17	8	19	11	27	10	12	3	256	0.68
Comamonadaceae	Polaromonas	0	1	0	0	58	79	22	38	10	19	6	9	4	4	1	2	2	1	255	0.67

Acidobacteria_Gp16	Gp16	27	11	17	10	11	2	11	6	20	10	22	12	25	9	18	7	21	10	250	0.66
OP10	OP10_genera_IS	13	6	18	10	19	7	13	4	19	10	17	5	28	17	24	6	21	10	246	0.65
Sphingomonadaceae	Sphingomonas	24	7	22	11	13	2	15	9	14	5	13	6	17	10	21	15	22	6	234	0.62
WS3	WS3_genera_IS	18	5	23	13	16	2	16	10	16	8	15	5	20	12	17	4	15	7	222	0.58
Alteromonadaceae	Haliea	11	4	6	2	8	5	9	3	17	11	12	4	29	19	16	3	32	26	216	0.57
Caulobacteraceae	Brevundimonas	8	6	12	5	14	8	13	7	11	3	11	6	22	11	24	19	25	8	211	0.56
Acidobacteria_Gp7	Gp7	19	5	20	12	19	7	18	9	14	7	16	6	14	7	13	7	11	4	207	0.55
Planctomycetaceae	Pirellula	23	13	10	5	8	4	8	5	18	15	19	4	14	8	14	7	22	10	206	0.54
Comamonadaceae	Acidovorax	1	1	13	13	4	6	39	27	15	8	15	10	3	5	11	10	4	4	188	0.50
Rhodocyclaceae	Propionivibrio	17	6	9	5	24	26	14	9	9	2	5	2	14	19	7	8	7	1	184	0.49
VerrucomicrobiaSD3	Subdivision3_gen_IS	10	6	24	31	17	12	9	4	10	11	6	3	5	5	10	7	9	5	184	0.49
Oxalobacteraceae	Massilia	23	13	7	4	7	4	14	7	15	6	13	5	16	15	10	6	11	5	183	0.48
Acidobacteria_Gp3	Gp3	13	7	18	12	15	8	14	4	13	7	9	3	16	10	12	4	7	3	174	0.46
Chloroplast	Cryptomonadaceae	1	1	15	12	23	22	17	10	3	5	15	9	7	3	9	9	2	4	169	0.45
Xanthomonadaceae	Lysobacter	11	5	11	4	9	4	8	4	17	9	14	4	12	7	13	5	14	6	156	0.41
Pseudomonadaceae	Pseudomonas	4	3	5	4	14	9	11	5	13	7	4	3	12	12	10	11	15	4	144	0.38
Chitinophagaceae	Flavisolibacter	14	5	14	7	7	2	9	7	12	8	8	2	5	3	6	6	6	2	123	0.32
Nocardioidaceae	Nocardioides	12	5	8	4	7	3	6	3	8	7	8	2	13	11	10	2	8	5	120	0.32
Chitinophagaceae	Lacibacter	20	8	12	3	10	6	9	4	7	4	5	2	5	3	7	3	6	3	116	0.31
Comamonadaceae	Ramlibacter	11	4	9	6	9	3	11	7	8	3	7	3	6	5	9	4	7	2	115	0.30
TM7	TM7_genera_IS	9	5	11	4	8	4	6	2	10	5	6	3	9	4	9	3	11	5	113	0.30
Xanthomonadaceae	Thermomonas	6	6	6	1	8	6	6	2	20	12	6	4	9	6	4	1	5	4	113	0.30
Comamonadaceae	Pelomonas	2	3	8	6	8	2	16	16	8	6	4	4	4	3	2	1	2	3	98	0.26
Acidimicrobidae_IS	Ilumatobacter	7	4	5	1	4	3	5	2	9	5	7	3	12	8	9	2	7	4	98	0.26
Porphyromonadaceae	Paludibacter	15	4	8	3	5	6	3	2	2	2	1	1	9	15	4	7	3	3	93	0.24
Burkholderiales_IS	Inhella	3	2	2	2	14	8	9	3	7	3	4	2	9	6	3	3	5	3	88	0.23
Micrococcaceae	Arthrobacter	11	4	6	3	4	2	3	2	9	6	7	2	4	1	4	5	7	5	86	0.23
Cytophagaceae	Leadbetterella	5	3	2	2	2	1	4	4	3	0	1	0	1	2	11	10	20	14	85	0.22
Erythrobacteraceae	Porphyrobacter	9	5	10	4	5	3	4	1	6	3	5	1	7	5	2	2	6	5	82	0.22
Planctomycetaceae	Rhodopirellula	8	4	3	2	2	1	2	1	6	5	5	4	7	7	4	3	10	5	78	0.21
Geobacteraceae	Geobacter	8	5	6	4	9	9	4	3	4	2	2	0	5	4	2	2	1	1	73	0.19
Nocardioidaceae	Marmoricola	9	5	5	3	4	1	3	3	5	4	3	1	5	6	4	2	6	3	72	0.19
Caulobacteraceae	Caulobacter	3	3	7	3	3	2	3	1	1	1	15	11	4	5	4	1	3	2	71	0.19
Cystobacteraceae	Anaeromyxobacter	8	3	6	4	5	2	4	3	4	1	4	2	7	3	4	3	5	2	70	0.18

Chitinophagaceae	Sediminibacterium	1	1	1	1	4	3	11	8	4	4	3	3	4	2	7	4	4	1	64	0.17
Caldilineaceae	Caldilinea	6	3	3	1	2	2	3	3	5	3	7	3	4	3	4	2	5	2	61	0.16
Oxalobacteraceae	Duganella	4	1	4	5	3	2	3	2	4	2	2	3	3	1	2	2	7	9	60	0.16
Pseudomonadaceae	Cellvibrio	0	0	0	0	2	2	5	3	3	2	2	2	10	11	4	5	5	2	59	0.16
Neisseriaceae	Vogesella	0	0	0	0	15	17	4	8	2	4	1	2	2	1	0	1	0	0	59	0.15
Methylococcaceae	Methylobacter	7	3	3	1	1	1	1	1	5	4	6	4	4	1	3	3	4	3	55	0.14
Acetobacteraceae	Roseomonas	3	2	4	3	2	2	3	2	3	2	5	3	6	2	3	1	5	3	52	0.14
Acidobacteria_Gp11	Gp11	4	3	4	2	3	1	3	2	3	2	5	3	3	1	4	3	4	2	52	0.14
Rhodocyclaceae	Zoogloea	2	3	2	2	1	1	1	0	1	1	10	11	4	4	1	2	3	1	49	0.13
Planctomycetaceae	Gemmata	3	1	3	3	3	1	2	1	3	3	6	4	6	2	3	2	3	1	49	0.13
Iamiaceae	Iamia	2	3	2	2	4	1	3	2	3	2	3	1	6	4	2	2	4	2	48	0.13
Burkholderiaceae	Limnobacter	0	0	10	10	3	5	1	3	1	1	5	4	1	2	1	1	0	1	48	0.13
Verrucomicrobiaceae	Prosthecobacter	6	5	2	2	3	4	2	2	1	1	1	1	2	2	3	3	5	3	47	0.12
Planctomycetaceae	Schlesneria	4	7	2	3	3	1	1	1	1	1	3	2	4	2	1	1	4	3	46	0.12
Verrucomicrobiaceae	Luteolibacter	7	6	3	2	2	2	1	1	3	3	1	2	2	1	2	2	1	1	44	0.12
Planctomycetaceae	Zavarzinella	4	3	2	2	2	1	1	1	3	2	4	3	3	2	1	1	3	1	42	0.11
Caulobacteraceae	Phenylobacterium	2	3	3	3	2	0	2	1	2	3	3	1	4	2	4	3	3	0	42	0.11
Polyangiaceae	Sorangium	2	2	3	2	1	2	2	1	4	3	4	1	2	1	3	2	3	2	39	0.10
Sphingobacteriaceae	Pedobacter	5	4	2	2	1	1	1	1	3	2	2	1	1	2	4	2	2	1	37	0.10
Comamonadaceae	Giesbergeria	0	1	0	0	8	9	2	5	1	2	1	1	0	1	0	0	1	2	35	0.09
Chromatiaceae	Rheinheimera	0	1	4	3	4	1	5	3	4	1	1	1	1	1	1	0	2	2	34	0.09
Chitinophagaceae	Niastella	6	5	2	2	3	1	3	1	3	1	1	0	1	1	1	0	1	1	33	0.09
SAR11	Pelagibacter	2	2	6	6	4	2	1	1	0	0	3	2	0	1	1	1	0	0	33	0.09
Polyangiaceae	Byssovorax	1	1	1	1	2	1	2	1	2	2	3	3	4	2	3	2	2	1	33	0.09
Aeromonadaceae	Aeromonas	2	1	2	1	3	4	3	3	2	2	1	1	1	1	0	1	2	1	33	0.09
Cytophagaceae	Adhaeribacter	2	2	1	1	2	2	2	1	2	2	3	2	1	0	2	2	4	2	33	0.09
Acidobacteria_Gp5	Gp5	2	1	2	2	1	1	3	3	1	2	2	1	2	2	3	2	2	0	32	0.08
Legionellaceae	Legionella	1	1	2	1	1	1	1	1	3	2	2	2	2	2	3	3	2	1	31	0.08
Microbacteriaceae	Cryobacterium	2	2	1	1	1	1	1	0	2	3	2	2	2	2	2	2	2	2	31	0.08
Bacillaceae	Exiguobacterium	6	2	2	2	2	1	2	1	2	0	1	1	0	0	2	2	2	1	30	0.08
Desulfobulbaceae	Desulfobulbus	4	2	3	1	3	2	2	1	2	1	1	1	2	2	1	0	1	1	30	0.08
Bradyrhizobiaceae	Balneimonas	3	2	1	1	1	1	2	2	4	2	2	1	1	1	2	1	2	2	30	0.08
Rhodobacteraceae	Rubellimicrobium	3	3	2	2	2	1	1	1	2	1	1	0	2	3	1	1	2	1	29	0.08
Saprospiraceae	Aureispira	0	1	5	4	5	1	3	3	2	1	1	1	1	1	0	0	0	0	29	0.08

Cyanobacteria_Family VI	GpVI	1	1	2	2	2	1	2	1	2	3	3	2	1	0	1	1	2	1	28	0.07
Oxalobacteraceae	Herbaspirillum	2	2	1	1	1	1	2	2	2	2	2	2	1	1	2	2	2	1	27	0.07
Planctomycetaceae	Planctomyces	2	2	1	1	2	1	1	0	2	2	2	1	1	0	1	1	3	2	27	0.07
Comamonadaceae	Comamonas	0	0	0	0	1	2	4	7	3	3	1	1	0	1	1	2	0	1	27	0.07
Comamonadaceae	Malikia	1	1	1	2	2	2	3	2	2	1	0	1	1	1	3	3	2	1	27	0.07
Burkholderiales_IS	Aquabacterium	0	1	1	1	1	0	1	1	5	5	4	2	1	1	1	0	0	0	26	0.07
Hyphomonadaceae	Hyphomonas	1	2	3	1	2	2	1	1	1	1	1	1	2	3	1	1	1	2	26	0.07
Burkholderiales_IS	Rubrivivax	2	2	3	1	2	0	2	1	2	1	2	1	2	1	2	1	2	1	26	0.07
Coxiellaceae	Aquicella	0	1	1	1	1	1	0	0	2	1	2	2	3	4	3	1	1	1	25	0.06
Chitinophagaceae	Parasegetibacter	2	2	1	1	1	1	1	1	1	1	2	1	2	1	2	1	2	1	23	0.06
OD1	OD1_genera_IS	0	1	2	2	2	1	2	3	1	1	1	2	1	1	1	1	1	1	23	0.06
Oxalobacteraceae	Undibacterium	0	0	0	0	2	2	2	1	1	1	0	0	1	1	3	4	3	1	23	0.06
Methylophilaceae	Methylotenera	0	1	2	3	1	1	1	1	0	1	2	2	1	1	2	2	1	1	22	0.06
Desulfobulbaceae	Desulforhopalus	2	2	1	1	1	1	1	1	1	1	2	2	2	1	2	1	1	0	22	0.06
Verrucomicrobiaceae	Haloferula	1	2	1	2	1	1	0	1	2	2	1	1	1	1	1	2	1	1	22	0.06
Thermaceae	Meiothermus	1	2	1	1	1	0	1	0	3	3	3	1	1	1	1	1	1	1	22	0.06
Hyphomicrobiaceae	Rhodoplanes	1	2	1	1	2	2	2	2	1	1	1	1	1	1	1	1	1	0	22	0.06
Acidobacteria_Gp18	Gp18	1	2	1	2	1	1	0	1	1	1	2	2	1	1	1	1	1	1	20	0.05
Bdellovibrionaceae	Bdellovibrio	2	2	0	1	2	1	1	1	1	1	0	0	0	0	1	2	1	1	20	0.05
Solirubrobacteraceae	Solirubrobacter	2	2	1	1	1	1	0	0	0	1	1	0	1	1	2	3	1	1	19	0.05
Erythrobacteraceae	Croceicoccus	1	2	1	1	0	0	0	0	0	0	2	2	3	2	1	1	1	1	19	0.05
Moraxellaceae	Acinetobacter	5	3	2	1	0	0	2	2	1	1	0	1	0	0	0	0	0	0	19	0.05
Alcaligenaceae	Azohydromonas	1	1	2	1	2	1	1	1	1	1	1	1	1	1	1	1	1	1	19	0.05
Trueperaceae	Truepera	2	1	1	1	1	1	1	0	1	1	1	1	1	1	1	0	1	2	19	0.05
Bradyrhizobiaceae	Bosea	1	2	2	1	0	1	1	1	1	1	1	1	0	0	2	2	1	1	18	0.05
Burkholderiaceae	Polynucleobacter	1	1	2	3	2	1	0	1	0	0	1	1	0	0	2	2	1	1	18	0.05
Microbacteriaceae	Microbacterium	0	0	0	0	0	0	1	1	1	1	1	0	0	0	3	3	4	3	17	0.05
Comamonadaceae	Pseudorhodoferax	0	1	1	2	1	1	0	0	0	1	1	1	2	2	1	1	1	1	17	0.05
